# Job Strain and Alcohol Intake: A Collaborative Meta-Analysis of Individual-Participant Data from 140 000 Men and Women

**DOI:** 10.1371/journal.pone.0040101

**Published:** 2012-07-06

**Authors:** Katriina Heikkilä, Solja T. Nyberg, Eleonor I. Fransson, Lars Alfredsson, Dirk De Bacquer, Jakob B. Bjorner, Sébastien Bonenfant, Marianne Borritz, Hermann Burr, Els Clays, Annalisa Casini, Nico Dragano, Raimund Erbel, Goedele A. Geuskens, Marcel Goldberg, Wendela E. Hooftman, Irene L. Houtman, Matti Joensuu, Karl-Heinz Jöckel, France Kittel, Anders Knutsson, Markku Koskenvuo, Aki Koskinen, Anne Kouvonen, Constanze Leineweber, Thorsten Lunau, Ida E. H. Madsen, Linda L. Magnusson Hanson, Michael G. Marmot, Martin L. Nielsen, Maria Nordin, Jaana Pentti, Paula Salo, Reiner Rugulies, Andrew Steptoe, Johannes Siegrist, Sakari Suominen, Jussi Vahtera, Marianna Virtanen, Ari Väänänen, Peter Westerholm, Hugo Westerlund, Marie Zins, Töres Theorell, Mark Hamer, Jane E. Ferrie, Archana Singh-Manoux, G. David Batty, Mika Kivimäki, for the IPD-Work Consortium

**Affiliations:** 1 Finnish Institute of Occupational Health, Helsinki, Finland; 2 Institute of Environmental Medicine, Karolinska Institutet, Stockholm, Sweden; 3 School of Health Sciences, Jönköping University, Jönköping, Sweden; 4 Department of Public Health, Ghent University, Ghent, Belgium; 5 National Research Centre for the Working Environment, Copenhagen, Denmark; 6 Inserm U1018, Centre for Research in Epidemiology and Population Health, Villejuif, France; 7 Versailles-Saint Quentin University, Versailles, France; 8 Department of Occupational Medicine, Bispebjerg University Hospital, Copenhagen, Denmark; 9 Centre for Maritime Health and Safety, Esbjerg, Denmark; 10 School of Public Health, Université Libre de Bruxelles, Brussels, Belgium; 11 Institute for Medical Informatics, Biometry, and Epidemiology, University Duisburg-Essen, Essen, Germany; 12 Department of Cardiology, West-German Heart Center Essen, University Duisburg-Essen, Essen, Germany; 13 TNO, Hoofddorp, The Netherlands; 14 Department of Health Sciences, Mid Sweden University, Sundsvall, Sweden; 15 Department of Public Health, University of Helsinki, Helsinki, Finland; 16 Warsaw School of Social Sciences and Humanities, Wroclaw Faculty, Wroclaw, Poland; 17 Stress Research Institute, Stockholm University, Stockholm, Sweden; 18 Department of Epidemiology and Public Health, University College London, London, United Kingdom; 19 Department of Occupational and Environmental Medicine, Bispebjerg University Hospital, Copenhagen, Denmark; 20 Department of Public Health and Clinical Medicine, Occupational and Environmental Medicine, Umeå University, Umeå, Sweden; 21 Finnish Institute of Occupational Health, Turku, Finland; 22 Department of Public Health and Department of Psychology, University of Copenhagen, Copenhagen, Denmark; 23 Department of Medical Sociology, University of Düsseldorf, Düsseldorf, Germany; 24 Folkhälsan Research Center, Helsinki, Finland; 25 Department of Public Health, University of Turku, Turku, Finland; 26 Turku University Hospital, Turku, Finland; 27 Occupational and Environmental Medicine, Uppsala University, Uppsala, Sweden; 28 School of Community and Social Medicine, University of Bristol, Bristol, United Kingdom; Catholic University of Sacred Heart of Rome, Italy

## Abstract

**Background:**

The relationship between work-related stress and alcohol intake is uncertain. In order to add to the thus far inconsistent evidence from relatively small studies, we conducted individual-participant meta-analyses of the association between work-related stress (operationalised as self-reported job strain) and alcohol intake.

**Methodology and Principal Findings:**

We analysed cross-sectional data from 12 European studies (n = 142 140) and longitudinal data from four studies (n = 48 646). Job strain and alcohol intake were self-reported. Job strain was analysed as a binary variable (strain vs. no strain). Alcohol intake was harmonised into the following categories: none, moderate (women: 1–14, men: 1–21 drinks/week), intermediate (women: 15–20, men: 22–27 drinks/week) and heavy (women: >20, men: >27 drinks/week). Cross-sectional associations were modelled using logistic regression and the results pooled in random effects meta-analyses. Longitudinal associations were examined using mixed effects logistic and modified Poisson regression. Compared to moderate drinkers, non-drinkers and (random effects odds ratio (OR): 1.10, 95% CI: 1.05, 1.14) and heavy drinkers (OR: 1.12, 95% CI: 1.00, 1.26) had higher odds of job strain. Intermediate drinkers, on the other hand, had lower odds of job strain (OR: 0.92, 95% CI: 0.86, 0.99). We found no clear evidence for longitudinal associations between job strain and alcohol intake.

**Conclusions:**

Our findings suggest that compared to moderate drinkers, non-drinkers and heavy drinkers are more likely and intermediate drinkers less likely to report work-related stress.

## Introduction

It has been hypothesised that stress in general and work-related stress in particular influence people's alcohol drinking habits and that work-related stress is associated with heavy drinking [1]. Bidirectional mechnisms for this association have been suggested. On one hand, people may use alcohol in an attempt to relieve stress at work [2], [3]; on the other hand, excessive alcohol intake is likely to reduce efficiency at work, thus possibly leading to or increasing work-related stress [4]. However, many other factors, such as genetic and epigenetic background as well as learned and cultural drinking behaviours, are likely to influence the relationship between alcohol intake and stress at work [5], [6]. Therefore, the extent to which work-related stress and alcohol intake are linked at population level remains unclear.

To date, the findings from epidemiological studies of the associations between alcohol intake and work-related stress have differed in direction and magnitude in subgroups of participants [7], [8], [9], [10], [11]. An important limitation of existing studies is that none thus far has been large enough to detect small or moderate associations or examine the associations of work-related stress and alcohol intake using high resolution categories of this behaviour. Public health guidelines in many countries suggest that consuming more than approximately 14 (for women) or 21 (for men) drinks per week confers significant risk to health [12], [13], [14], [15]. However, findings from recent meta-analyses suggest that the threshold for harmful drinking may be somewhat higher, with some adverse health effects of alcohol consumption becoming evident in women and men who drink more than approximately 20 or 27 drinks a week, respectively [16], [17], [18]. However, it is currently not known what threshold of alcohol intake is related, or relevant, to work stress.

In order to better understand the relationship between alcohol drinking and stress at work, we undertook individual-participant meta-analyses of the association between work-related stress and alcohol intake. We used a large set of data pooled from 12 independent European studies with a common measure of work stress, job strain [1], [19]. In this large dataset, we were able to split alcohol intake into more categories than previous studies and examine the associations of alcohol intake and work stress in socio-demographic subgroups.

## Methods

### Studies and Participants

We conducted individual-level meta-analyses using pooled data from 12 independent studies conducted between 1985 and 2008 in Belgium, Denmark, Finland, France, Germany, the Netherlands, Sweden and the UK. The studies are a part of the “Individual-participant-data Meta-analysis of Working Populations” (IPD-Work) Consortium, which was established at the annual Four Centers Meeting in London, November 8, 2008. A pre-defined two-stage data acquisition protocol was used. The first stage involved the acquisition and harmonisation of baseline data on work stress as well as socio-demographic and lifestyle factors. The second stage involves the acquisition and analysis of disease outcomes. Our meta-analyses were based on the first stage cross-sectional data and were thus conducted before any linkage to disease data.

Details of the design and participants of the studies included in our meta-analyses have been published previously [20] and are described in Appendix S1. Data from the following studies were used: Belstress, Danish Work Environment Cohort Study (DWECS), Finnish Public Sector Study (FPS), Health and Social Support (HeSSup), Heinz Nixdorf Recall study (HNR), Intervention Project on Absence and Well-being (IPAW), Permanent Onderzoek Leefsituatie (POLS), Burnout, Motivation and Job Satisfaction study (Danish acronym PUMA), Whitehall II and Work Lipids and Fibrinogen (WOLF) Norrland and Stockholm. Participants with complete data on job strain, alcohol intake, age, sex and socioeconomic position were included in the analyses (Table 1).

**Table 1:** Participant and study summary.

Study^1^ (country)	N participants^2^	N (%) female	Age: mean (SD) range	N (%) with job strain	% none/moderate/intermediate/heavy drinkers^2^
Belstress (Belgium)	20 732	4 850 (23.4)	45.5 (5.9) 33–61	3 880 (18.7)	19.1/59.0/7.4/14.5
DWECS (Denmark)	5 564	2 603 (46.8)	41.8 (11.0) 18–69	1 236 (22.2)	56.0/38.4/0/5.6
FPS (Finland)	45 807	36 990 (80.8)	44.6 (9.4) 17–65	7 384 (16.1)	13.6/76.1/3.9/6.4
Gazel (France)	11 052	3 021 (27.3)	50.3 (3.0) 43–58	1 590 (14.4)	12.0/69.7/7.1/11.2
HeSSup (Finland)	16 431	9 121 (55.5)	39.6 (10.2) 43–58	2 876 (17.5)	13.7/76.4/5.0/5.0
HNR (Germany)	1 798	736 (40.9)	53.4 (5.0) 45–73	218 (12.2)	20.7/59.4/6.4/13.4
IPAW (Denmark)	1 981	1 318 (66.5)	41.2 (10.5) 18–68	343 (17.3)	17.1/76.0/3.9/3.0
POLS (the Netherlands)	16 548	5 949 (35.9)	39.1 (11.4) 15–85	2548 (15.4)	8.0/83.7/7.2/1.1
PUMA (Denmark)	1 807	1 490 (82.5)	42.6 (10.2) 18–69	273 (15.1)	16.9/76.9/4.4/1.9
Whitehall II (United Kingdom)	10 285	3 399 (33.1)	44.4 (6.1) 34–56	1 441 (14.0)	19.0/65.4/5.6/9.9
WOLF Norrland (Sweden)	4 597	760 (16.5)	44.0 (10.3) 19–65	585 (12.7)	6.4/87.6/2.9/3.1
WOLF Stockholm (Sweden)	5 538	2 402 (43.4)	41.5 (11.0) 19–70	886 (16.0)	3.8/87.9/3.5/4.8
**All**	142 140	72 629 (51.1)	44.0 (8.7), 15–85	23 260 (16.4)	14.5/72.4/5.1/8.0

SD: standard deviation.

1 Study acronyms: DWECS: Danish Work Environment Cohort Study; FPS: Finnish Public Sector Study; HeSSup: Health and Social Support; HNR: Heinz Nixdorf Recall study; IPAW: Intervention Project on Absence and Well-being; POLS: Permanent Onderzoek Leefsituatie; PUMA: Burnout, Motivation and Job Satisfaction study; WOLF: Work Lipids and Fibrinogen. ^2^ Participants with complete data on job strain, age, sex and socioeconomic position.

2 Moderate drinking (women: 1–14 drinks/week, men: 1–21 drinks/week); intermediate drinking (women: 15–20 drinks/week, men: 22–27 drinks/week); heavy drinking (women: > = 21 drinks/wk, men: > = 28 drinks/week).

We estimated cross-sectional associations between alcohol intake and work stress in 12 studies comprising 142 140 individuals (mean age: 44.0 years). In addition, the associations of alcohol intake and job strain in socio-demographic subgroups were investigated in studies in which we had access to individual-level data (n = 116 240; Figure S1). Longitudinal associations of alcohol intake and job strain were examined using individual-level repeated measurements data from four studies, in which these data were available (n = 48 646; Figure S1).

### Ethical Approval

Each constituent study in the IPD-Work consortium was approved by the relevant local or national ethics committees and all participants gave informed consent to take part. Details of the ethical approval are provided in Appendix S1.

### Ascertainment of Alcohol Intake

Alcohol intake was ascertained from questions on the total number of alcoholic drinks, by type of drink, which the participants consumed in a week. One drink was defined as approximately equivalent to one unit or one glass of alcoholic drink or 10 g of ethanol. Participants were categorised according to their alcohol intake as follows: non-drinkers, moderate drinkers (women: 1–14 drinks/week, men: 1–21 drinks/week), intermediate drinkers (women: 15–20 drinks/week, men: 22–27 drinks/week) and heavy drinkers (women: > = 21 drinks/wk, men: > = 28 drinks/week). The lower limit for intermediate drinking is the upper limit of healthy alcohol consumption according to public health guidelines in many countries [12], [13], [14], [15]. The lower limit for heavy drinking is based on an empirically estimated threshold at which the beneficial health effects of alcohol turn to an increased risk adverse effects, such as type-2 diabetes, liver cirrhosis and death [16], [17], [18], [21]. Previous analyses in the FPS (the largest study in the current meta-analysis) showed that in this study individuals reporting heavy drinking had 4.27 (95% CI: 2.55. 7.15) times as high 5-year risk of death from alcohol-related causes, compared to those who did not report heavy alcohol use [22].

### Ascertainment of Work Stress

Work stress was operationalised as job strain and defined as high job demands and low job control [1]. Job strain was ascertained in all studies using sets of questions from the Job Content Questionnaire (JCQ) and Demand-Control Questionnaire (DCQ) [1], [19]. A description of the questionnaires and job demand and job control scales in the IPD-Work Consortium studies is provided elsewhere [23]. Mean response score for job demands items and mean response score for job control items were calculated for each participant. Job strain was defined as having a high demands score (>study-specific median score) and a low control score (<study-specific median score). All other combinations of job demands and job control, including the values equal to the median value, were assigned to the no strain category. Participants with missing data on more than half of the job demands or job control items (n = 1 523, 1%) were excluded.

### Covariates

Information on sex and age was obtained from population registries or baseline interview with the participant (in DWECS, FPS, Gazel, HNR, IPAW, PUMA, WOLF Norrland and WOLF Stockholm) or from a participant-completed questionnaire (in Belstress, HeSSup, POLS and Whitehall II). Sex was modelled as a binary and age as a continuous variable (years). Socioeconomic position was defined based on occupational title obtained from employers' or other registries (in DWECS, FPS, Gazel, IPAW and PUMA) or participant-completed questionnaires (in Belstress, HeSSup, HNR, POLS, Whitehall II, WOLF Norrland and WOLF Stockholm). In HeSSup socioeconomic position was defined based on the participant's self-reported highest educational qualification. Socioeconomic position was categorised in all studies as low (e.g. cleaners, maintenance workers), intermediate (e.g. registered nurses, technicians) or high (e.g. teachers, physicians). Participants who were self-employed or who had missing data on job title were included in the analyses in the “other” socioeconomic position category (n = 1 109, 0.8%).

### Statistical Analyses

We used individual-level data provided by the investigators in Belstress, FPS, Gazel, HeSSup, HNR, Whitehall II, WOLF Norrland and WOLF Stockholm studies. Some groups of investigators (in DWECS, IPAW, POLS and PUMA) undertook the statistical analyses themselves according to instructions from the IPD-Work analytical team and provided the IPD-Work team with the study-specific results.

For our main analyses, we used a two-stage meta-analytical approach because we had access to individual-level data from eight studies and aggregate data from four studies (DWECS, IPAW, POLS and PUMA). Logistic regression models were used to estimate study-specific associations between alcohol intake and job strain alcohol intake and the resulting estimates and their standard errors were pooled using fixed effect and random effects meta-analyses [24], [25], [26]. We quantified heterogeneity in the effect estimates using the I^2^ statistic, which indicates the proportion of the total variation in the estimates that is due to between-studies variation [27]. In addition, we used a one-stage meta-analytical approach, pooling the available individual-level data into one dataset, to investigate exposure-covariate interactions and longitudinal associations because this approach provides a flexible way of investigating individual-level interactions [26], [28], [29], [30]. One-stage analyses were conducted using mixed effects logistic regression, with the study as the random effect. Interactions were investigated by stratifying the models for sex, age group and socioeconomic position, and tested by including an interaction term (alcohol*covariate) in the model that also contained the main effects. Longitudinal associations were examined using mixed effects logistic regression with study as the random effect when the outcomes were rare, and modified Poisson regression with robust standard errors and study as the cluster-variable when the outcomes were not rare [31].

All meta-analyses and statistical analyses in the pooled individual-level data were performed using Stata SE 11.0 (Stata Corporation, College Station, Texas, USA). In POLS the study-specific analyses were undertaken by the study team using SPSS 17 (SPSS Inc., Chicago, Illinois, USA) and in, DWECS, IPAW and PUMA using SAS 9 (SAS Institute Inc., Cary, North Carolina, USA).

## Results

### Alcohol Intake and Job Strain

The characteristics of participants included in our analyses are shown in Table 1. Of the 142 140 participants, 23 260 (16.4%) reported job strain, 20 547 (14.5%) were non-drinkers, 102 905 (72.4%) moderate drinkers, 7 299 (5.1%) intermediate drinkers and 11 389 (8.0%) heavy drinkers.

**Figure 1:**
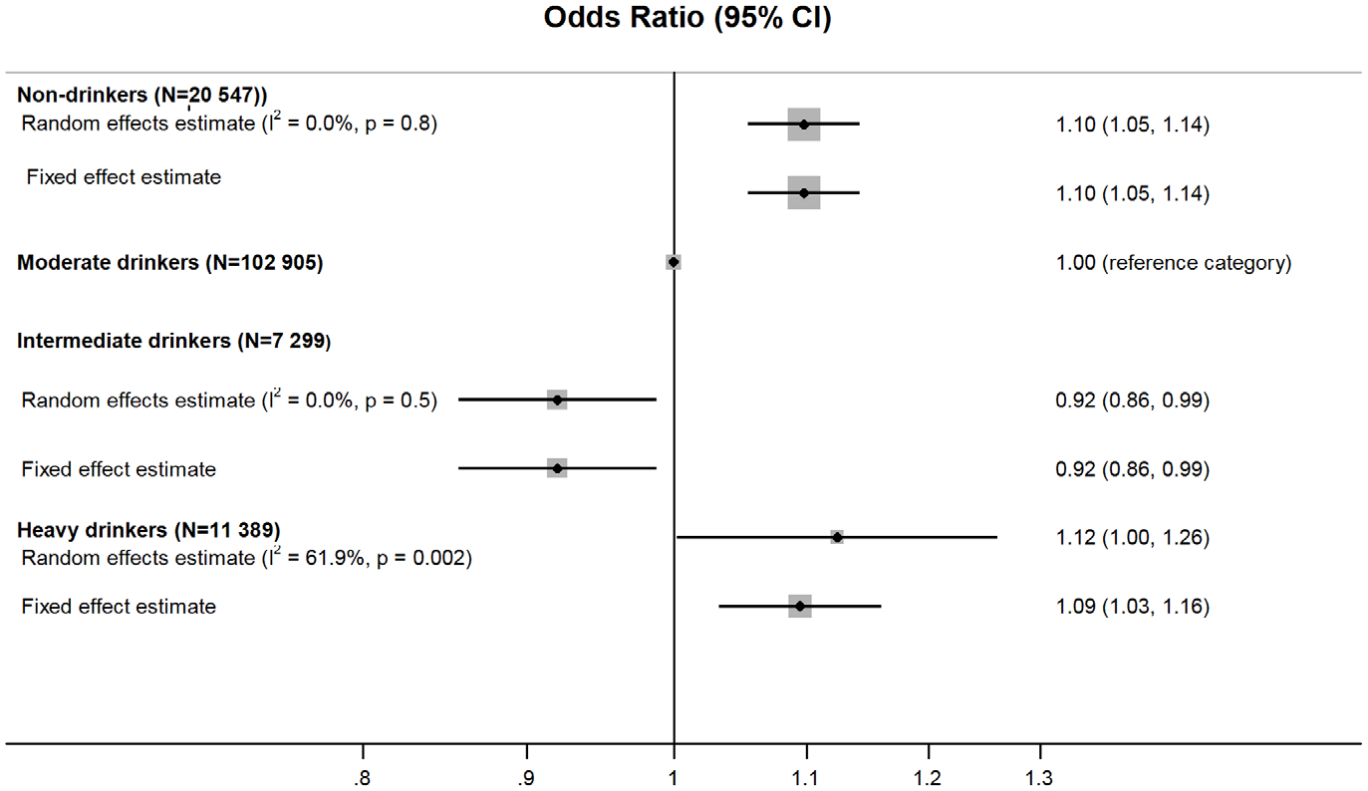
Association of alcohol intake and job strain (adjusted for age, sex and socioeconomic position) (N = 142 140).

A meta-analysis of alcohol intake and job strain is shown in Figure 1. Random effects meta-analyses were considered to be the most appropriate approach and the results from these will be described throughout this article, though fixed effect estimates are shown in figures for comparison. Compared to moderate drinkers, non-drinkers had, on average, higher odds of job strain (age, sex and socioeconomic position -adjusted random effects odds ratio (OR): 1.10, 95% CI: 1.05, 1.14). Intermediate drinkers (those consuming more alcohol than is advised healthy in many countries but less than what is associated with the risk of severe disease outcomes), on the other hand, had lower odds of job strain than moderate drinkers (random effects OR: 0.92, 95% CI: 0.86, 0.99). Compared to moderate drinkers, the odds of job strain in heavy drinkers were higher (random effects OR: 1.12, 95% CI (1.00, 1.26). We observed little heterogeneity in the effect estimates among non-drinkers and intermediate drinkers and the random effects and fixed effect ORs in these groups were identical or nearly identical. By contrast, the effect estimates among heavy drinkers were heterogeneous (I^2^∶61.9%). The study-specific effect estimates are shown in Figure S2. We explored whether additional adjustment for tobacco smoking influenced our effect estimates in the studies in which we had access to individual-level data. However, the results additionally adjusted for tobacco smoking were nearly identical to our main findings: the random effects ORs for job strain among non-, intermediate and heavy drinkers were 1.10 (95% CI: 10.5, 1.15), 0.93 (95% CI: 0.84, 1.01), and 1.10 (95% CI: 1.00, 1.21), respectively.

We investigated access to individual-level data in the IPD-Work Consortium as a possible source of heterogeneity by stratifying our meta-analyses by the availability of individual-level data (Figure S3). Overall, the pooled effect estimates from the studies in which we had no access to individual-level data were less precisely estimated than those from the studies which had these data available but differences in the effect sizes were generally small.

### Stratified Analyses in Socio-demographic Subgroups

The findings from the meta-analysis stratified by demographic covariates (sex, age and socioeconomic position) and based on individual-level data are shown in Table S1. The association of intermediate drinking with job strain were similar in men and women but the associations between non-drinking and heavy drinking with job strain were stronger in men than in women. The lower odds of job strain in intermediate drinkers were particularly prominent in intermediate drinkers aged 50 or older. The odds of job strain varied according to socioeconomic position but we observed no clear pattern in this variation.

### Longitudinal Analyses of Alcohol Intake and Job Strain

Longitudinal analyses were based on pooled data with repeated measurements from Belstress, FPS, HeSSup and Whitehall II. The longitudinal associations between job strain at baseline and taking up excessive drinking (i.e. becoming an intermediate or heavy drinker) by follow-up among baseline non-drinkers and moderate drinkers are presented in Table 2. The associations of excessive drinking at baseline and job strain at follow-up (among baseline excessive drinkers) are shown in Table 3. There was no clear evidence for associations between either job strain at baseline and taking up excessive drinking by follow-up or excessive drinking at baseline and job strain at follow-up. Associations of alcohol intake at baseline and job strain at follow-up, stratified by baseline job strain, are shown in Table 4. Generally, alcohol intake at baseline was not associated with moving from the no strain to the strain category or vice versa between baseline and follow-up. However, compared to baseline moderate drinkers, baseline non-drinkers who reported no strain at baseline were more likely to develop job strain by follow-up (OR for strain at follow-up: 1.14, 95% CI: 1.04, 1.25).

**Table 2:** Longitudinal associations between job strain and taking up excessive drinking^1^ among baseline moderate and non-drinkers (n = 43 665)^2^.

Exposure	N participants	N (%) taking up excessive drinking^1^	OR (95% CI)^3^ for taking up excessive drinking by follow-up
Job strain at baseline			
No	36 389	2 130 (5.8)	1 (reference category)
Yes	6 976	332 (4.8)	0.90 (0.79, 1.01)
Job strain at baseline and follow-up			
No and no	32 867	1 973 (5.9)	1 (reference category)
No and yes	3 822	187 (4.9)	0.89 (0.76, 1.01)
Yes and no	4 117	197 (4.8)	0.87 (0.75, 1.01)
Yes and yes	2 859	135 (4.7)	0.91 (0.76. 1.09)

1 Excessive drinker: an individual who drinks more than recommended amounts of alcohol (intermediate or heavy drinker).

2 Studies and follow-up times: Belstress (4–7 years), FPS (2–4 years), HeSSup (5 years) and Whitehall II (3–9 years.).

3 Odds ratios (ORs) from a mixed effects logistic model, adjusted for baseline age, sex and baseline socioeconomic position, with study as the random effect.

**Table 3:** Longitudinal associations between job strain and reducing alcohol intake to moderate or no alcohol, among baseline excessive drinkers (n = 4 981)^12^.

Exposure	N participants	N (%) reducing alcohol intake	OR (95% CI)^2^ reducing alcohol intake
Job strain at baseline			
No	4 321	1 689 (39.1)	1 (reference category)
Yes	660	273 (41.4)	1.91 (0.76, 1.08)
Job strain at baseline and follow-up			
No and no	3 951	1 520 (38.5)	1 (reference category)
No and yes	370	169 (45.7)	1.15 (0.93, 1.43)
Yes and no	424	174 (41.0)	0.94 (0.76, 1.15)
Yes and yes	236	99 (42.0)	0.89 (0.68, 1.18)

1 Excessive drinker: an individual who drinks more than recommended amounts of alcohol (intermediate or heavy drinker).

2 Studies and follow-up times: Belstress (4–7 years), FPS (2–4 years), HeSSup (5 years) and Whitehall II (3–9 years.).

3 Odds ratios (ORs) from a mixed effects logistic model, adjusted for baseline age, sex and baseline socioeconomic position, with study as the random effect.

**Table 4:** Associations of alcohol intake at baseline and job strain at follow-up, stratified by baseline job strain (n = 48 646)^1^.

Population by baseline exposure	N participants	N (%) developing job strain	OR (95% CI)^2^ for job strain at follow-up
No job strain at baseline (N = 41 010)			
Non-drinker	5 503	713 (13.0)	1.14 (1.04, 1.25)
Moderate drinker	31 186	3 109 (10.0)	1 (reference category)
Intermediate drinker	1 731	143 (8.3)	0.94 (0.79, 1.12)
Heavy drinker	2 590	227 (8.8)	0.97 (0.84, 1.12)
		**N (%) no job strain at follow-up**	**IRR (95% CI)** 3 **for no job strain at follow-up**
Job strain at baseline (N = 7 636)			
Non-drinker	1 275	715 (56.1)	0.97 (0.91 1.03)
Moderate drinker	5 701	3 402 (59.7)	1 (reference category)
Intermediate drinker	247	159 (64.4)	1.06 (1.00, 1.13)
Heavy drinker	413	265 (63.2)	1.07 (1.01, 1.13)

1 Studies and follow-up times: Belstress (4–8 years), FPS (2–4 years), HeSSup (5 years) and Whitehall II (3–9 years).

2 Odds ratios (ORs) from a mixed effects logistic model, adjusted for baseline age, sex and baseline socioeconomic position, with study as the random effect.

3 Incidence rate ratios (IRRs) from a modified Poisson model, adjusted for baseline age, sex and baseline socioeconomic position, with robust standard errors and study as the cluster variable.

## Discussion

In our pooled analyses of data from 12 European studies, individuals who reported abstaining from alcohol were 10% more likely to experience job strain than those who reported drinking moderate amounts. Compared to moderate drinkers, intermediate drinkers were 8% less likely and heavy drinkers were 12% more likely to report job strain, although the latter association was imprecisely estimated.

Our finding that non-drinkers were more likely to report job strain than moderate drinkers is difficult to interpret because the non-drinkers are a heterogeneous group. In addition to lifelong abstainers who choose not to drink alcohol for religious or conscience reasons, this group is likely to include individuals who do not drink alcohol because of previous alcohol addiction or ill health, both of which may also be associated with work-related stress. Further research would be needed to examine the role of these factors. There are several biological mechanisms that could explain the observed lower odds of job strain in intermediate drinkers (individuals drinking more than recommended amounts of alcohol but less than heavy drinkers) when compared to moderate drinkers. Ethanol can produce positive mood states with stress-relieving effects through the activation of the endocannabinoid system CB1 receptors and µ/ð-opioid receptors [3]. A similar mechanism could explain the heavy drinkers' higher odds of job strain when compared to moderate drinkers', if heavy alcohol intake is an attempt to relieve work-related stress [3] or if reduced efficiency at work, relating to heavy alcohol intake, induces the experience of work-related stress [4]. However, the cross-sectional associations observed in our meta-analyses were generally small and there was no clear evidence of longitudinal associations between alcohol intake and work stress.

The strengths of our investigation were that it was based on a large set of individual-level data, and as far as we are aware, is the largest study of alcohol intake and work stress to date. Thus, we were able to provide more precise estimates of the association between job strain and alcohol intake than has been possible before [7], [8], [9], [10], [11]. A major advantage of individual-participant meta-analysis of published as well as unpublished data, such as ours, is that it usually provides a larger sample size than individual studies and minimises publication bias, which can influence the findings of literature-based meta-analyses [32]. Work stress was defined in all studies using a widely used and accepted measure, job strain [19]. However, the job strain measure has been criticised as a measure of stress because it is based on assessment of the attributes of the job (demands and control) and not direct questions on whether the respondent experiences these or other aspects of their job as stressful. The participants' alcohol intake was self-reported in all the studies and it is likely that some misclassification of alcohol intake has influenced our findings, as under-reporting of alcohol intake is common in population-based studies [33], [34] and might be particularly frequent among heavy drinkers. Also, non-drinkers in our analyses included both lifetime abstainers and individuals who have drunk alcohol previously but stopped drinking by the time they participated in the study. Thus, some misclassification of alcohol intake may have influenced our study-specific estimates of the association between work stress and alcohol intake, and introduced heterogeneity to our meta-analyses. It is also possible that some of our findings have been influenced by residual confounding from unmeasured confounders, such as mood disorders, personality, addiction, or proneness to addictive behaviour [35], [36], [37], [38]. Also, our findings were based on European participants, which may limit their generalisability to other populations.

### Conclusions

Our findings suggest that compared to moderate drinkers, non-drinkers and heavy drinkers are more likely to report work stress. Intermediate drinkers (individuals who drink more than moderate amounts but not excessively so) were less likely to report work stress than moderate drinkers. We found no clear evidence for longitudinal associations between self-reported alcohol intake and work stress.

## Supporting Information

Figure S1 **Studies and participants included in the analyses.**
(DOC)

Figure S2 **Associations of alcohol intake and job strain (adjusted for age, sex and socioeconomic position).**
(DOC)

Figure S3 **Associations of alcohol use and job strain, stratified by the availability of individual-level data (adjusted for age, sex and socioeconomic position).**
(DOC)

Table S1 **Associations of alcohol intake and work job strain in demographic subgroups^1^.**
(DOC)

Appendix S1 **Study design and recruitment of participants in the 12 European studies included in our individual-participant meta-analyses of alcohol intake and work stress.**
(DOC)
