# Supplementary material for: Job Strain and Alcohol Intake: A Collaborative Meta-Analysis of Individual-Participant Data from 140 000 Men and Women
Source: PLoS One. 2012 Jul 6;7(7):e40101. doi: 10.1371/journal.pone.0040101 (PMC3391232; doi:10.1371/journal.pone.0040101)
Supplement: Figure S1 — Studies and participants included in the analyses. (DOC) [file pone.0040101.s001.doc]

**Figure S1. Studies and participants included in the analyses**

**IPD Work meta-analysis:**

12 studies: Belstress, DWECS, FPS, Gazel, HeSSup, HNR, IPAW, POLS, PUMA, Whitehall II WOLF Norrland and WOLF Stockholm.

**Meta-analyses of alcohol use and work stress:**

12 studies: Belstress, DWECS, FPS, Gazel, HeSSup, HNR, IPAW, POLS, PUMA, Whitehall II WOLF Norrland and WOLF Stockholm

N=142 140

**Individual-level pooled data**

(analyses of alcohol and work stress in subgroups):

8 studies: Beltress, FPS, Gazel, HeSSup, HNR, Whitehall II, WOLF Norrland and WOLF Stockholm.

N=116 240

**Individual-level pooled data with repeated measurements** (repeated measures analyses of alcohol use and work stress):

4 studies: Belstress, FPS, HeSSup and Whitehall II.

N=48 646
