# Supplementary material for: Job Strain and Alcohol Intake: A Collaborative Meta-Analysis of Individual-Participant Data from 140 000 Men and Women
Source: PLoS One. 2012 Jul 6;7(7):e40101. doi: 10.1371/journal.pone.0040101 (PMC3391232; doi:10.1371/journal.pone.0040101)
Supplement: Figure S2 — Associations of alcohol intake and job strain (adjusted for age, sex and socioeconomic position). (DOC) [file pone.0040101.s002.doc]

**Figure S2. Associations of alcohol intake and job strain (adjusted for age, sex and socioeconomic position)**

NOTE: Weights are from random effects analysis

.

.

.

.

**Non-drinkers (n=20 547)**

Belstress

DWECS

FPS

Gazel

HNR

HeSSup

IPAW

POLS

PUMA

WOLF Norrland

WOLF Stockholm

Whitehall

Random effects estimate (I-squared = 0.0%, p = 0.8)

Fixed effect estimate

**Moderate drinkers** (reference category) **n=102 905**

**Intermediate drinkers (n=7 299)**

Belstress

FPS

Gazel

HNR

HeSSup

IPAW

POLS

PUMA

WOLF Norrland

WOLF Stockholm

Whitehall

DWECS

Random effects estimate (I-squared = 0.0%, p = 0.5)

Fixed effect estimate

**Heavy drinkers (n=11 389)**

Belstress

DWECS

FPS

Gazel

HNR

HeSSup

IPAW

POLS

PUMA

WOLF Norrland

WOLF Stockholm

Whitehall

Random effects estimate (I-squared = 61.9%, p = 0.002)

Fixed effect estimate

Study

3957

3115

6237

1325

376

2252

338

180

305

294

212

1956

1542

1776

789

116

818

78

1195

79

134

192

580

0

3010

311

2922

1240

240

814

60

1330

34

142

265

1021

drinkers

Number of

19.09

55.98

13.62

11.99

20.91

13.71

17.06

1.09

16.91

6.40

3.83

19.02

7.44

3.88

7.14

6.45

4.98

3.94

7.22

4.38

2.91

3.47

5.64

0.00

14.52

5.59

6.38

11.22

13.35

4.95

3.03

8.04

1.88

3.09

4.79

9.93

drinkers

%

1.18 (1.08, 1.29)

1.02 (0.89, 1.18)

1.08 (1.01, 1.16)

1.01 (0.86, 1.18)

1.11 (0.79, 1.57)

1.17 (1.05, 1.31)

1.11 (0.82, 1.51)

1.03 (0.88, 1.21)

1.20 (0.85, 1.69)

1.13 (0.80, 1.59)

1.03 (0.72, 1.48)

1.05 (0.91, 1.22)

1.10 (1.05, 1.14)

1.10 (1.05, 1.14)

0.98 (0.85, 1.14)

0.97 (0.83, 1.12)

0.98 (0.78, 1.23)

0.47 (0.20, 1.11)

0.74 (0.60, 0.93)

0.85 (0.44, 1.65)

0.87 (0.73, 1.03)

1.24 (0.67, 2.31)

0.81 (0.46, 1.42)

0.92 (0.61, 1.39)

0.94 (0.73, 1.22)

(Excluded)

0.92 (0.86, 0.99)

0.92 (0.86, 0.99)

1.20 (1.08, 1.34)

1.14 (0.85, 1.54)

0.92 (0.82, 1.04)

1.07 (0.89, 1.29)

1.13 (0.70, 1.82)

1.22 (1.02, 1.46)

0.94 (0.45, 1.96)

0.88 (0.58, 1.31)

4.18 (2.01, 8.71)

1.32 (0.82, 2.11)

1.22 (0.88, 1.68)

1.03 (0.85, 1.25)

1.12 (1.00, 1.26)

1.09 (1.03, 1.16)

OR (95% CI)

19.64

8.02

32.24

6.32

1.34

12.60

1.75

6.54

1.37

1.36

1.21

7.61

100.00

24.34

23.32

9.72

0.69

10.35

1.16

16.91

1.31

1.56

2.97

7.66

0.00

100.00

15.27

8.13

14.90

12.03

4.35

12.15

2.14

5.52

2.14

4.42

7.34

11.61

100.00

(D+L)

Weight

%

1.18 (1.08, 1.29)

1.02 (0.89, 1.18)

1.08 (1.01, 1.16)

1.01 (0.86, 1.18)

1.11 (0.79, 1.57)

1.17 (1.05, 1.31)

1.11 (0.82, 1.51)

1.03 (0.88, 1.21)

1.20 (0.85, 1.69)

1.13 (0.80, 1.59)

1.03 (0.72, 1.48)

1.05 (0.91, 1.22)

1.10 (1.05, 1.14)

1.10 (1.05, 1.14)

0.98 (0.85, 1.14)

0.97 (0.83, 1.12)

0.98 (0.78, 1.23)

0.47 (0.20, 1.11)

0.74 (0.60, 0.93)

0.85 (0.44, 1.65)

0.87 (0.73, 1.03)

1.24 (0.67, 2.31)

0.81 (0.46, 1.42)

0.92 (0.61, 1.39)

0.94 (0.73, 1.22)

(Excluded)

0.92 (0.86, 0.99)

0.92 (0.86, 0.99)

1.20 (1.08, 1.34)

1.14 (0.85, 1.54)

0.92 (0.82, 1.04)

1.07 (0.89, 1.29)

1.13 (0.70, 1.82)

1.22 (1.02, 1.46)

0.94 (0.45, 1.96)

0.88 (0.58, 1.31)

4.18 (2.01, 8.71)

1.32 (0.82, 2.11)

1.22 (0.88, 1.68)

1.03 (0.85, 1.25)

1.12 (1.00, 1.26)

1.09 (1.03, 1.16)

19.64

8.02

32.24

6.32

1.34

12.60

1.75

6.54

1.37

1.36

1.21

7.61

100.00

24.34

23.32

9.72

0.69

10.35

1.16

16.91

1.31

1.56

2.97

7.66

0.00

100.00

15.27

8.13

14.90

12.03

4.35

12.15

2.14

5.52

2.14

4.42

7.34

11.61

100.00

(D+L)

Weight

%

1

.3

1

6

D+L: DerSimonian and Laird
