# Supplementary material for: Job Strain and Alcohol Intake: A Collaborative Meta-Analysis of Individual-Participant Data from 140 000 Men and Women
Source: PLoS One. 2012 Jul 6;7(7):e40101. doi: 10.1371/journal.pone.0040101 (PMC3391232; doi:10.1371/journal.pone.0040101)
Supplement: Figure S3 — Associations of alcohol use and job strain, stratified by the availability of individual-level data (adjusted for age, sex and socioeconomic position). (DOC) [file pone.0040101.s003.doc]

**Figure S3. Associations of alcohol use and job strain, stratified by the availability of individual-level data (adjusted for age, sex and socioeconomic position)**

.

.

.

.

.

.

.

**Non-drinkers, no individual-level data**

Random effects estimate (I^2^ = 0.0%, p = 0.8)

Fixed effect estimate

**Non-drinkers, with individual-level data**

Random effects estimate (I^2^ = 0.0%, p = 0.6)

Fixed effect estimate

**Intermediate drinkers, no individual-level data**

Random effects estimate (I^2^ = 0.0%, p = 0.5)

Fixed effect estimate

**Intermediate drinkers, with individual-level data**

Random effects estimate (I^2^ = 8.2%, p = 0.4)

Fixed effect estimate

**Heavy drinkers, no individual-level data**

Random effects estimate (I^2^ = 78.5%, p = 0.003)

Fixed effect estimate

**Heavy drinkers, with individual-level data**

Random effects estimate (I^2^= 51.9%, p = 0.04)

Fixed effect estimate

1.05 (0.95, 1.15)

1.05 (0.95, 1.15)

1.11 (1.06, 1.16)

1.11 (1.06, 1.16)

0.89 (0.75, 1.04)

0.89 (0.75, 1.04)

0.92 (0.85, 1.01)

0.93 (0.86, 1.00)

1.33 (0.78, 2.26)

1.17 (0.94, 1.45)

1.10 (1.00, 1.22)

1.09 (1.03, 1.16)

OR (95% CI)

1.05 (0.95, 1.15)

1.05 (0.95, 1.15)

1.11 (1.06, 1.16)

1.11 (1.06, 1.16)

0.89 (0.75, 1.04)

0.89 (0.75, 1.04)

0.92 (0.85, 1.01)

0.93 (0.86, 1.00)

1.33 (0.78, 2.26)

1.17 (0.94, 1.45)

1.10 (1.00, 1.22)

1.09 (1.03, 1.16)

1

.442

1

2.26

Age, sex and SEP-adjusted OR for job strain
