# Supplementary material for: Job Strain and Alcohol Intake: A Collaborative Meta-Analysis of Individual-Participant Data from 140 000 Men and Women
Source: PLoS One. 2012 Jul 6;7(7):e40101. doi: 10.1371/journal.pone.0040101 (PMC3391232; doi:10.1371/journal.pone.0040101)
Supplement: Table S1 — Associations of alcohol intake and work job strain in demographic subgroups1. (DOC) [file pone.0040101.s004.doc]

**Table S1. Associations of alcohol intake and work job strain in demographic subgroups1**

|  | **N (n with job strain) % with job strain** | **OR for job strain (95% CI)^2^** |
| --- | --- | --- |
| **Non-drinkers (n=16 609) vs. moderate drinkers (n=84 030)** | | |
| All | 16 609 (3 322) 20.0% | 1.11 (1.06, 1.16) |
| Stratified by sex |  |  |
| Men | 5 885 (961) 16.3 % | 1.21 (1.13, 1.32) |
| Women | 10 724 (2 361) 22.0 % | 1.04 (0.99, 1.10) |
| p for interaction ^3^ |  | 0.05 |
| Stratified by age |  |  |
| <50 years | 10 551 (2 067) 19.6 % | 1.11 (1.05, 1.17) |
| >=50 years | 6 058 (1 255) 20.7 % | 1.09 (1.01, 1.17) |
| p for interaction ^3^ |  | 0.7 |
| Stratified by socioeconomic position |  |  |
| Low | 5 253 (1 307) 24.9 % | 1.12 (1.04, 1.21) |
| Intermediate | 8 667 (1 793) 20,7 % | 1.10 (1.04, 1.17) |
| High | 2 539 (189) 7.4 % | 1.12 (0.96, 1.32) |
| p for interaction ^3^ |  | 0.006 |
| **Intermediate drinkers (n=5 947) vs. moderate drinkers (n=84 030)** | | |
| All | 5 947 (805) 13.5% | 0.93 (0.86, 1.00) |
| Stratified by sex |  |  |
| Men | 3 951 (463) 11.7 % | 0.96 (0.87, 1.07) |
| Women | 1 996 (342) 17.1 % | 0.91 (0.81, 1.03) |
| p for interaction ^3^ |  | 0.8 |
| Stratified by age |  |  |
| <50 years | 3 737 (552) 14.8 % | 0.99 (0.90, 1.08) |
| >=50 years | 2 210 (253) 11.5 % | 0.83 (0.73, 0.96) |
| p for interaction ^3^ |  | 0.005 |
| Stratified by socioeconomic position |  |  |
| Low | 1 271 (255) 20.1 % | 1.04 (0.90, 1.20) |
| Intermediate | 2 797 (441) 15.8 % | 0.88 (0.79, 0.97) |
| High | 1 815 (100) 5.5 % | 0.85 (0.69, 1.05) |
| p for interaction ^3^ |  | 0.2 |
| **Heavy drinkers (n=9 654) vs. moderate drinkers (n=84 030)** | | |
| All | 9 654 (1 523) 15.8% | 1.09 (1.03, 1.16) |
| Stratified by sex |  |  |
| Men | 6 560 (959) 14.6 % | 1.17 (1.08, 1.26) |
| Women | 3 094 (564) 18.2 % | 1.02 (0.93, 1.13) |
| p for interaction ^3^ |  | 0.1 |
| Stratified by age |  |  |
| <50 years | 6 141 (1 020) 16.6 % | 1.11 (1.03, 1.19) |
| >=50 years | 3 513 (503) 14.3 % | 1.07 (0.96, 1.18) |
| p for interaction ^3^ |  | 0.1 |
| Stratified by socioeconomic position |  |  |
| Low | 2 246 (460) 20.5 % | 1.07 (0.96, 1.20) |
| Intermediate | 4 541 (857) 18.9 % | 1.09 (1.01, 1.18) |
| High | 2 719 (180) 6.6 % | 1.02 (0.87, 1.21) |
| p for interaction ^3^ |  | 0.4 |

^1^ In pooled set of individual-level data from eight studies: Beltress, FPS, Gazel, HeSSup, HNR, Whitehall II, WOLF Norrland and WOLF Stockholm (N=127 556).

^2^ Odds ratios from a mixed effects logistic model with job strain as the outcome, drinking category as the main exposure, age, sex and socioeconomic position as covariates (where appropriate) and study as the random effect.

^3^ p-value for alcohol use*covariate.
